# Role of Reduced Protein Intake in Metabolic and Healthspan Benefits of Plant-Based Diets

**DOI:** 10.1093/geroni/igaa057.2624

**Published:** 2020-12-16

**Authors:** James Mitchell, Michael MacArthur, Sarah Mitchell

**Affiliations:** 1 ETH Zurich, Zurich, Massachusetts, Switzerland; 2 Harvard T.H. Chan School of Public Health, Boston, Massachusetts, United States

## Abstract

Plant-based nutrition consisting of vegetarian/vegan dietary patterns are positively associated with metabolic fitness and inversely associated with risk of cardiovascular disease, diabetes and all-cause mortality. The nutritional and molecular basis of such benefits remains unclear. Here we considered the potential contribution of protein quality (amino acid profiles) and quantity to plant-based nutritional benefits. To this end, we investigated whether individuals adhering to plant-based diets consume a different AA profile, and used isocaloric diets in controlled rodent studies with modulation of both AA composition and total protein amount using crystalline AA vs. naturally-sourced protein ingredients. We found surprisingly few differences between AA profiles of vegans vs. omnivores, but large effects of total protein independent of source in rodent studies, strongly suggesting a major effect of total protein rather than AA composition in health benefits of plant-based diets. Mechanistically, we discuss the role of reduced protein intake on glucose and lipid homeostasis.

